# The Seasonal and Spatial Patterns of Planktonic Bacterial and Fungal Community Structure and the Response to Environmental Factors in the Danjiangkou Reservoir, China

**DOI:** 10.1111/1758-2229.70362

**Published:** 2026-05-13

**Authors:** Yarui Cheng, Xiaoyu Lu, Zunwei Ke, Lu Gan, Yaqing Zhou, Fengjian Wang

**Affiliations:** ^1^ Shiyan Key Laboratory of Biological Resources and Eco‐Environmental Protection, College of Chemical and Environmental Engineering Hanjiang Normal University Shiyan China

**Keywords:** community assembly, Danjiangkou Reservoir, dry and wet season, environmental factors, planktonic microorganisms

## Abstract

Planktonic microbial communities are essential for sustaining the cycling of materials and the flow of energy. However, little research has examined how planktonic bacteria and fungi in the Danjiangkou Reservoir (DJKR) are distributed and function across different seasons. The findings indicated that the DJKR maintained generally favourable water quality. Seasonal changes have a more pronounced effect on fungal diversity than on bacterial diversity, resulting in a significant decrease in fungal diversity. Among bacterioplankton, the dominant phyla were Proteobacteria, Actinobacteria and Bacteroidetes, while Basidiomycota, Ascomycota, Mucoromycota and Chytridiomycota accounted for 81.22% of the total fungi, being the dominant fungal groups. According to the KEGG annotation, the most active functions in bacteria and fungi were amino acid metabolism and translation, respectively. Seasonal shifts exerted a notable effect on the abundance of genes participating in nitrogen and phosphorus cycles. Total nitrogen (TN), pH, total phosphorus (TP) and water temperature (WT) were identified by redundancy analysis (RDA) as the key environmental factors influencing the community structure of bacteria and fungi. Stochastic processes exerted a predominant influence on the assembly dynamics of planktonic bacterial and fungal communities. This study offers a scientific foundation for protecting water resources at the DJKR.

## Graphical abstract

This study investigated the spatial and temporal distribution patterns of bacterial and fungal communities in the Danjiangkou Reservoir during dry season and wet season, revealing the mechanism by which seasonal variations drive changes in microbial community composition and function through their influence on the physicochemical properties of the water.

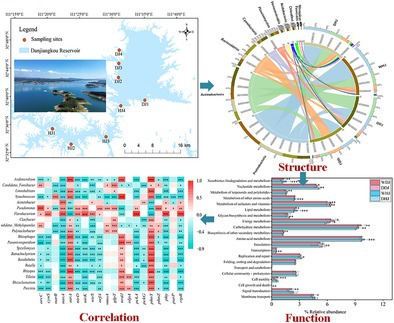

## 1 Introduction

The Danjiangkou Reservoir (DJKR) is Asia's largest artificial freshwater lake and supplies water for the Middle Route of China's South‐to‐North Water Diversion Project, which includes the Han river and Dan river (Liu et al. 2018). While the water quality of the DJKR has stayed at a low nutrient level, the normal water level has been elevated from 157 to 170 m due to dam construction. The DJKR has achieved its maximum water transfer capacity. This dynamic water transport might modify microbial community structure, consequently impacting the stability of aquatic ecosystems (Wang, Mao, et al. 2023). Human activities, pollutants in the environment and seasons can all cause changes in water properties. Thus, monitoring the water quality of DJKR is crucial.

In aquatic ecosystems, microorganisms are the dominant components of biological communities. Their diverse assemblages are essential for regulating biogeochemical processes, including nitrogen fixation, carbon decomposition and phosphorus solubilization (Pinhassi et al. 2022). Aquatic microorganisms are the first response barrier to water health and sensitivity to environmental changes, so regular monitoring of water quality can be achieved through changes in the diversity, interactions and functions of microbial communities. The way aquatic microorganisms are structured and interact with each other was considered a key metric for the biological evaluation and water quality monitoring in reservoirs (Mohapatra et al. 2020). The environmental factors (e.g., pH, water‐dissolved oxygen, temperature and the contents of phosphorus and nitrogen nutrients) and biotic factors (predation and competition) can both influence aquatic microbial communities (Hu et al. 2017). Environmental changes can lead to oxidative stress in aquatic microorganisms, which in turn increases the antioxidant enzymes activity, including superoxide dismutase (SOD), catalase and malondialdehyde (Zhou et al. 2024). Nevertheless, few studies have been conducted on aquatic microorganisms antioxidant enzymes activity and water quality evaluation in DJKR.

The ecological safety of water resources in DJKR has been highlighted by many current studies. Therefore, a more thorough knowledge of microbial community composition and ecological traits within DJKR is required. Since the majority of aquatic microorganisms are unculturable, high‐throughput sequencing has become a key method for analysing the composition and function of water microbial communities (Keneally et al. 2025). According to high‐throughput sequencing analysis, the planktonic bacterial community in DJKR contained 27 phyla, whereas the fungal community contained 7 phyla. Environmental factors (notably TN and pH) exerted a significant influence on the distribution of both communities (Chen et al. 2018; Chen, Xu, et al. 2020). Aquatic microorganisms may also exhibit yearly and seasonal fluctuations. The microeukaryotes algae in Ting River exhibit contrasting community compositions between wet and dry seasons, which could be attributed to the seasonal succession patterns of planktonic microeukaryotes (Chen et al. 2019). There was also a clear spatiotemporal pattern in bacterial communities in Karst Rivers, which was more affected by seasonal fluctuations than spatial fluctuations, and this appears to be influenced by seasonal environmental factors such as temperature and turbidity (Wu et al. 2022). DJKR experiences a dry season from February to July and a wet season from August to January of the subsequent year. However, few studies have been carried out on the influence of seasonal variations on the construction of microbial communities (Yang et al. 2023).

Recently, the mechanisms underlying community assembly have become a key focus in ecological studies. The study of microbial community assembly has utilized network analysis and model inference, the latter of which has been extensively adopted in aquatic ecosystems (Li, Jin, et al. 2023). The assembly patterns of microbial communities were shaped by both deterministic and stochastic ecological processes (Dini‐Andreote et al. 2015). In the Yellow River Basin, deterministic processes were found to dominate the assembly of fungal communities, with homogeneous selection playing the largest role (Fang et al. 2025). In high‐altitude Yarlung Zangbo river, studies have also revealed that with decreasing elevation, the role of deterministic processes intensifies in structuring fungal communities while diminishing for bacterial communities, with their assemblies being predominantly governed by homogeneous selection (Hao et al. 2025). Research conducted in DJKR on planktonic eukaryotes communities and how they assemble demonstrated that environmental fluctuations can cause a transition in community assembly from stochastic to deterministic processes (Zhu et al. 2025). A previous study has also determined the water depth variations influenced microbial community construction and symbiotic network stability in DJKR, which indicated bacteria exhibited a higher sensitivity response compared to fungi and other eukaryotes (Wang, Wang, Li, et al. 2025). Therefore, exploring how bacterial and fungal communities assemble across different seasons and spatial zones in the DJKR holds significant practical importance for sustainable water resource management of this critical aquatic system.

This study focuses on the DJKR, employing metagenomic sequencing technology to investigate the differences in distribution patterns of bacterial and fungal communities between dry and wet seasons, together with how environmental factors shape microbial community structure. The principal targets of this research are: (1) seasonal variations in the compositional and distributional characteristics of aquatic microbial communities (bacteria and fungi); (2) relationships between environmental factors (water quality parameters) and nutrient‐cycling functional genes with planktonic microbial communities; (3) characterizing seasonal differences in planktonic microbial community assembly dynamics between Hanjiang Reservoir (HJ) and Danjiang Reservoir (DJ). The findings may enhance understanding of the seasonal variations and assembly processes governing aquatic microbial communities in the DJKR, thereby providing a scientific basis for using aquatic microorganisms as indicators of reservoir water quality changes.

## 2 Materials and Methods

### 2.1 Field Sites and Sample Preparation

The DJKR is an important water resource for over 20 cities in China, which include the HJ and DJ. In this survey, 48 samplings were selected from 8 sites; the HJ and DJ each include 4 sites (Figure S1). Water samples were collected from two seasons of each site to investigate seasonal characteristics: February 2023 (dry season) from HJ and DJ (DHJ and DDJ), October 2023 (wet season) from HJ and DJ (WHJ and WDJ). From the surface water (0–50 cm) at each site, three replicate samples were obtained. Planktonic microorganisms were collected by filtering 1 L of water from each sample through a 0.22 μm microporous filter. The filter was then stored at −80°C for subsequent DNA extraction.

We measured water temperature (WT) and dissolved oxygen (DO) on site with a dissolved oxygen analyser (model JPBJ‐609L). The pH was detected by a portable pH meter (PHBJ‐261L). Water samples for chemical analysis were preserved at 4°C and analysed within 1 week. The determination of physicochemical properties of water followed the environmental quality standard for surface water of China (GB3838‐2002) (Chen, Liu, et al. 2022). Total nitrogen (TN) was determined using the alkaline potassium persulfate digestion UV spectrophotometric method, in which potassium persulfate converts both organic and inorganic nitrogen compounds into nitrate. Total phosphorus (TP) was determined by reacting with acidified molybdate to produce reduced phosphomolybdic blue, and the absorbance was measured spectrophotometrically at 700 nm. The Nessler's reagent spectrophotometric method was employed to determine ammonia nitrogen (NH_4_^+^—N). We measured superoxide dismutase (SOD) activity using the Total SOD assay kit with WST‐8 (catalogue no. S0101, Beyotime Biotechnology, Shanghai, China), following the steps described in the instruction manual. The microplate reader was used to measure the absorbance at 450 nm. Planktonic microbial SOD activity was standardized against total protein.

### 2.2 Extraction of DNA and Subsequent Sequencing

The genomic DNA of planktonic microorganisms was extracted from the membrane filters using the TGuide S96 magnetic bead soil/faecal genomic DNA extraction Kit (BioMarker, China), in accordance with the manufacturer's manual. The concentration and purity of the extracted DNA were measured using a NanoDrop2000 instrument (Thermo Fisher Scientific, Wilmington, DE), while DNA quality was assessed on 1% agarose gels. The library was prepared with the VAHTSTM Universal Plus DNA Library Prep Kit for Illumina (ND617‐02) following the manufacturer's protocol. We used Qsep‐400 to inspect fragment quality and Qubit 3.0 to quantify library concentration. For the constructed library, the Illumina Novassq 6000 sequencing platform was used for double‐ended sequenced, with a sequencing strategy of PE150.

### 2.3 Bioinformatics Analysis

The processing of raw sequencing data involves various steps. Raw tags were filtered using Fastp software to generate high‐quality sequencing data. We used Bowtie2 software to align reads with the host genome sequence and remove host contamination, obtaining clean reads. The MEGAHIT software was utilized for all cleaned sequences assembly and discarding contigs under 300 bp, and subsequently, QUAST software was employed to assess the assembly results (Gurevich et al. 2013; Li et al. 2015). The gene sequences in contigs were predicted by MetaGeneMark software (Version 3.26). Redundancy removal was performed using MMseqs2 software under thresholds of 95% similarity and 90% coverage. Functional annotation of genes was derived by aligning the protein sequences of non‐redundant genes against the NCBI NR database and the Kyoto Encyclopedia of Genes and Genomes (KEGG) database. From the KEGG database, we selected gene families associated with the nitrogen and phosphorus cycles. The species composition and relative abundance of the sample were derived from the alignment of non‐redundant genes against the NR database. The relative abundance of species was then calculated at the phylum and genus levels.

Alpha diversity reflects the richness and diversity of species and function, and it was evaluated using the Chao1 and Shannon indices. The β‐diversity of planktonic microorganisms among samples was assessed using non‐metric multidimensional scaling (NMDS) with bray‐curtis distance dissimilarities. The differential KEGG functional pathways were based on relative abundances and processed by *z*‐score normalization. Canoco 5 software was conducted to redundancy analysis (RDA), which evaluates the correlation between planktonic microorganisms and environmental factors. Using neutral model analysis, we assessed the contributions of stochastic and deterministic processes to microbial community assembly, where *R* ^2^ indicates model fit and ‘*m*’ signifies migration rate.

### 2.4 Statistical Analysis

The raw data was pre‐processed (calculation of mean and standard deviation) by Excel 2017 software. The significance of hydrochemical parameters and microbial functional gene abundances among different sample groups was calculated using IBM SPSS Statistics 19.0. The graphics were drawn employing Origin 2024.

## 3 Results

### 3.1 Water Quality and Microbial SOD Activity Assessment in the DJKR

**FIGURE 1:**
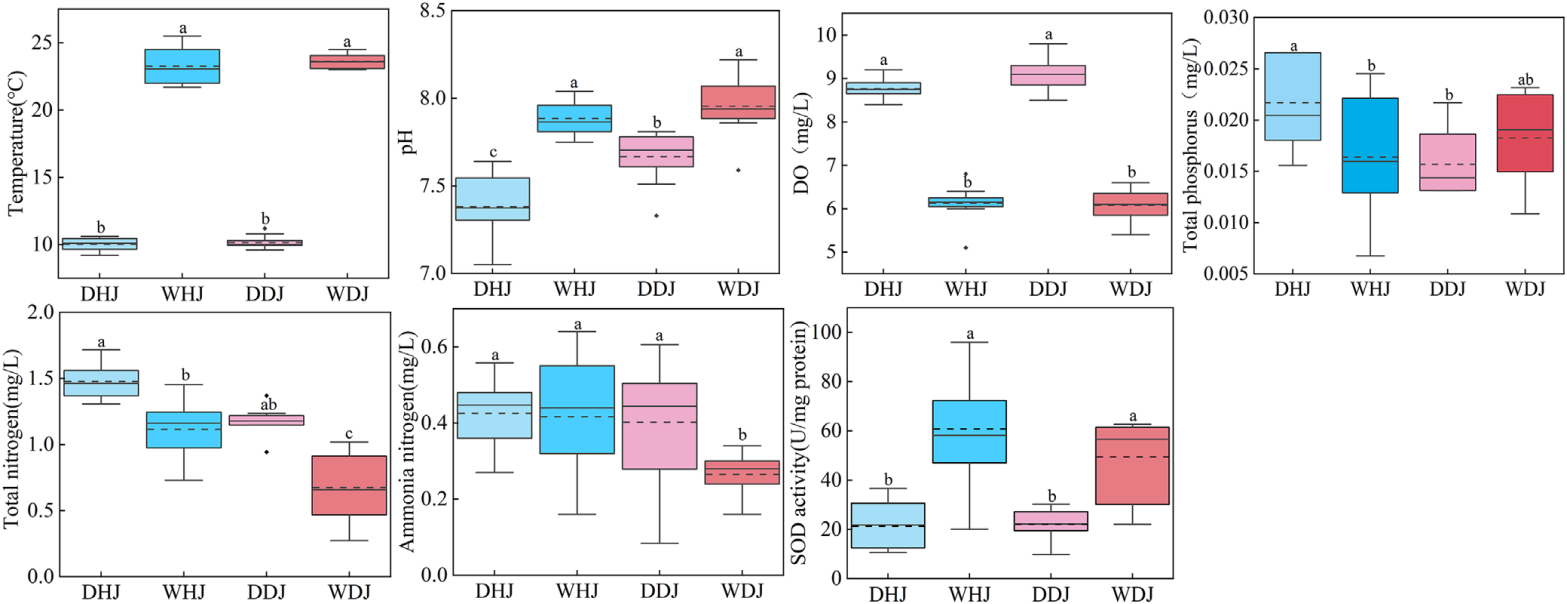
Physicochemical parameters of DJ and HJ water bodies during dry season and wet season. The presence of distinct lowercase letters indicates that the differences between groups are significant (*p* < 0.05).

During both seasons (dry and wet), the water quality of the DJKR generally remains satisfactory. Except for TN, all parameters conformed to the Class II water quality requirements set by China's GB38382‐2002 (Figure 1). We measured TN concentrations of 1.48 mg/L at DHJ, 1.11 mg/L at WHJ and 1.14 mg/L at DDJ, respectively, corresponding to Class IV surface water standards. In contrast, the average TN concentration at WDJ was 0.67 mg/L, which aligns with Class III surface water standards. A significant difference (*p* < 0.05) in TN levels was found between WDJ and the other monitoring sites through statistical analysis.

The trophic status of DJKR significantly influences the oxidative stress status of aquatic microorganisms. The spatial variations showed no significant effects on SOD activity in microbial communities (Figure 1). However, compared to the dry season at DJ and HJ, SOD activity in aquatic microorganisms during the wet season demonstrated a pronounced increase (*p* < 0.05). This phenomenon may be explained by the lower levels of DO, TN and NH_4_^+^‐N during the wet season, which potentially enhances microbial oxidative stress under oligotrophic conditions.

### 3.2 Seasonal Changes in Microbial Community Diversity and Composition in DJ and HJ

**FIGURE 2:**
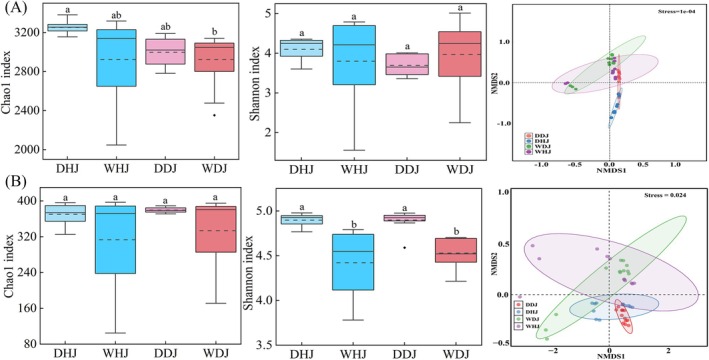
The α‐ and β‐diversities of bacteria and fungi in HJ and DJ. (A) The Chao1, Shannon and NMDS analysis of bacteria. (B) The Chao1, Shannon and NMDS analysis of fungi. The presence of distinct lowercase letters indicates that the differences between groups are significant (*p* < 0.05).

To evaluate microbial community richness and diversity in DJKR, the Chao1 index and Shannon index were applied in both seasons (dry and wet). Bacterioplankton communities exhibited no significant seasonal variation in either Chao1 or Shannon indices; the bacterioplankton diversity ranked as follows: DHJ > WDJ > WHJ > DDJ (Figure 2A). The Chao1 index of fungal communities showed no significant difference between the DJ and HJ across seasons, whereas seasonal variation significantly impacted the diversity of fungal communities in DJ and HJ (Figure 2B). This suggests that fungal community diversity was more susceptible to seasonal changes than that of bacterial community diversity. Two‐dimensional scatter plots from NMDS were presented in Figure 2, the stress values for NMDS ordination of planktonic bacterial and fungal communities were 0.0001 and 0.024, respectively. Planktonic microbial communities exhibited certain variations across different periods and locations, with planktonic fungal communities showing more distinct separation compared to bacterial communities.

**FIGURE 3:**
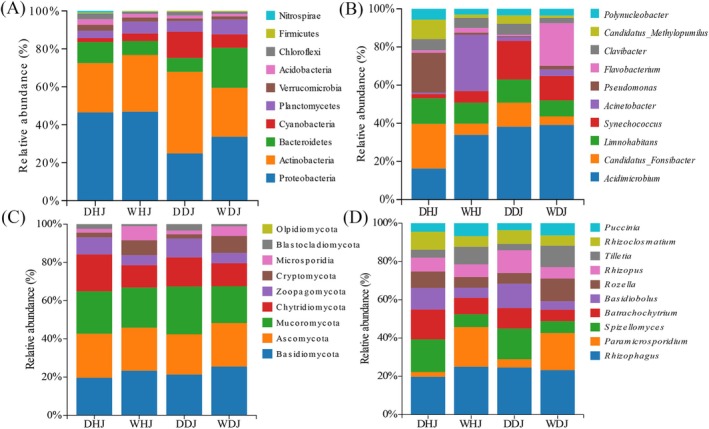
Bar chart depicting the relative abundances of bacterial and fungal communities at different levels. (A) The relative abundances of bacteria at the phylum level. (B) The relative abundances of bacteria at the genus level. (C) The relative abundances of fungi at the phylum level. (D) The relative abundances of fungi at the genus level.

The bacterioplankton community comprised 152 phyla and 2783 genera. Relative abundance histograms identified the most prevalent phyla and genera in the DHJ, DDJ, WHJ and WDJ samples. Proteobacteria, Actinobacteria and Bacteroidetes ranked as the top three phyla in terms of abundance (Figure 3A). Proteobacteria exhibited a higher relative abundance during the wet season relative to the dry season in DJ, and its abundance in HJ was higher than that in DJ (DHJ: 46.47%, WHJ: 46.88%, DDJ: 24.86%, WDJ: 33.69%). Actinobacteria relative abundance slightly increased in HJ but decreased in DJ during the wet season versus the dry season (DHJ: 26.03%, WHJ: 29.93%; DDJ: 43.02%, WDJ: 25.83%). Bacteroidetes abundance in HJ was lower in the wet season than in the dry season, whereas in DJ it increased by approximately threefold (DHJ: 11.05%, WHJ: 7.28%; DDJ: 7.31%, WDJ: 21.10%). At the genus level, *Acidimicrobium*, *Candidatus_Fonsibacter*, *Synechococcus* and *Pseudomonas* were the most abundant genera during the dry season (DHJ and DDJ). In contrast, *Acidimicrobium*, *Acinetobacter* and *Flavobacterium* dominated at wet season sampling sites (WHJ and WDJ) (Figure 3B). *Acidimicrobium* was more abundant at all sites except DHJ (DHJ: 16.26%, WHJ: 33.91%; DDJ: 38.19%, WDJ: 39.12%). Conversely, both *Candidatus_Fonsibacter* and *Pseudomonas* demonstrated higher abundances in DHJ compared to other sampling sites.

The planktonic fungal community primarily comprised 9 phyla and 465 genera. Basidiomycota, Ascomycota, Mucoromycota and Chytridiomycota collectively accounted for 81.22% of the total fungal population, representing the dominant fungal groups. The relative abundance of Basidiomycota increased in both HJ and DJ during the wet season (DHJ: 19.60%, WHJ: 23.33%, DDJ: 21.32%, WDJ: 25.49%), whereas Zoopagomycota and Chytridiaceae showed varying degrees of decline. *Rhizophagus* dominated the fungal community at the genus level. *Paramicrosporidium* showed an increase in relative abundance in the wet season, rising approximately 8‐fold in HJ and 4‐fold in DJ (DHJ: 2.54%, WHJ: 20.74%, DDJ: 4.34%, WDJ: 19.40%). Conversely, *Spizellomyces*, *Batrachochytrium* and *Basidiobolus* showed reduced abundances to varying extents during the wet season. In summary, the bacterial and fungal communities in DJ and HJ were differentially affected by seasonal variations.

### 3.3 Variations in Microbial Community Functional Redundancy Between HJ and DJ

**FIGURE 4:**
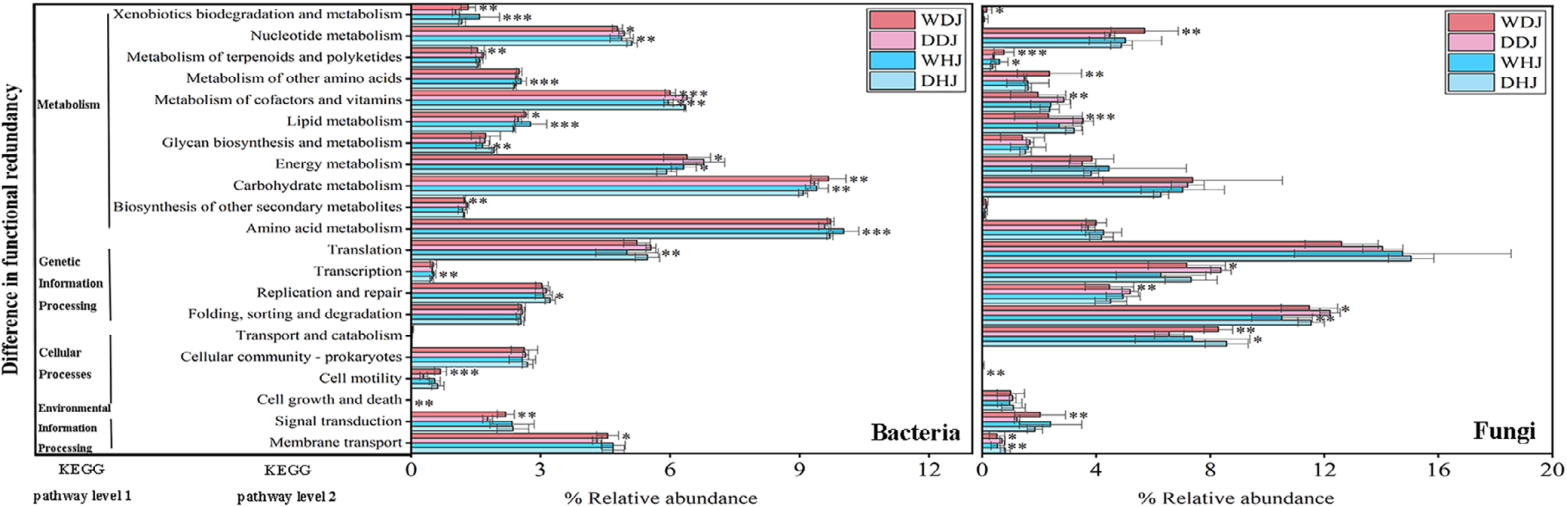
Column charts display differences in bacterial functions and fungal functions at KEGG level 2 between dry season and wet season. Asterisks above columns and close to numerical values denote significance: Wet season versus dry season (*p < 0.05; **p < 0.01; ***p < 0.001).

Changes in the microbial community would further lead to alterations in the overall metabolic functions of the planktonic microbial community. In bacterial KEGG level 2, the relative abundance of global and overview maps exceeded 30% (Figure S2), while amino acid metabolism, metabolism of cofactors and vitamins and energy metabolism were all present at relatively high levels (> 5%) (Figure 4). Compared to the dry season, significantly more bacterial functional pathways changed significantly during the wet season and even more changed significantly in the HJ (Figure 4). During the wet season, the bacterial functions as carbohydrate metabolism, lipid metabolism and xenobiotics biodegradation and metabolism showed a significant increase (*p* < 0.05, *p* < 0.01 or *p* < 0.001), while nucleotide metabolism and metabolism of cofactors and vitamins exhibited a significant decrease (*p* < 0.05, *p* < 0.01 or *p* < 0.001). Additionally, HJ showed significant elevations in bacterial functions including amino acid metabolism and metabolism of other amino acids (*p* < 0.001).

At KEGG level 2 for fungi, global and overview maps accounted for more than 20% of the relative abundance (Figure S2), while folding, sorting and degradation, transcription, translation and carbohydrate metabolism were all relatively abundant (> 5%) (Figure 4). Compared to the dry season, a significantly greater number of fungal functional pathways changed in DJ than in HJ during the wet season (Figure 4). In the wet season, we observed significant reductions in fungal functions of membrane transport and of folding, sorting and degradation (*p* < 0.05 or *p* < 0.01), while terpenoid and polyketide metabolism showed a significant increase (*p* < 0.05 or *p* < 0.001). Additionally, in the wet season of DJ, the fungal functions signal transduction, transport and catabolism, metabolism of other amino acids and nucleotide metabolism were significantly elevated (*p* < 0.01), while lipid metabolism and metabolism of cofactors and vitamins were significantly reduced (*p* < 0.01 or *p* < 0.001). These results highlight that seasonal variation significantly shaped the functional profiles of both bacterial and fungal communities, as well as the distinct seasonal responses of bacterial and fungal functions between the HJ and DJ.

### 3.4 Correlation Analysis of the Antioxidant Genes and Planktonic Microorganisms

**FIGURE 5:**
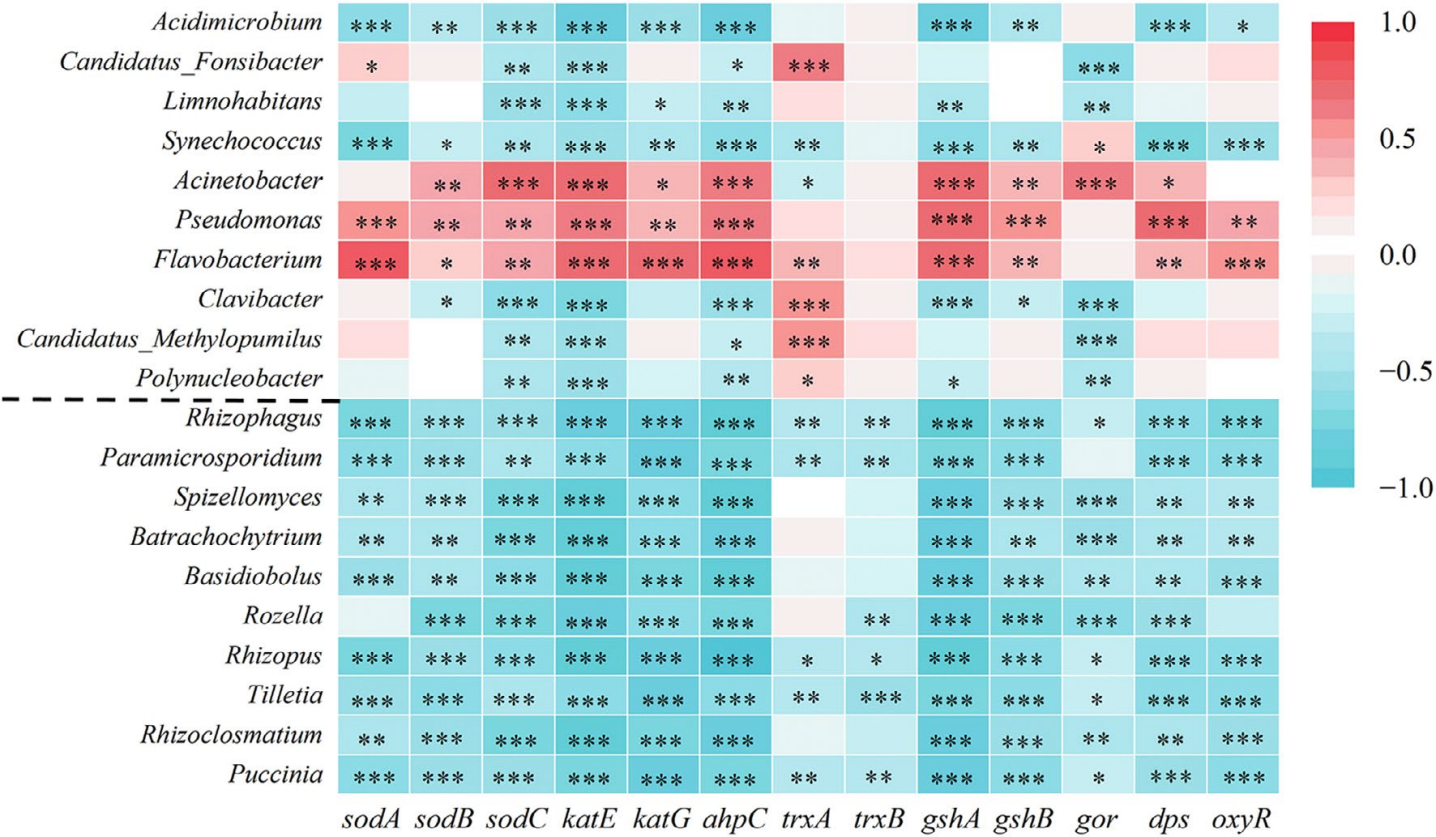
Heat map of Pearson correlation analysis of the antioxidant genes and bacterial (top 10) and fungal (bottom 10) at genus level. Asterisks above columns and close to numerical values denote significance (*p < 0.05; **p < 0.01; ***p < 0.001).

The antioxidant‐related genes abundance in DJ and HJ are shown in Table S1 and Figure S3, and higher abundances were observed in HJ compared to DJ. Most antioxidant genes exhibited no significant changes in the wet season relative to the dry season. In WDJ, the genes *katE* (catalase) and *ahpC* (alkyl hydroperoxide reductase C) abundance exhibited a significant increase, while a significant decrease in *trxB* (thioredoxin reductase) abundance was observed (*p* < 0.05, *p* < 0.01 or *p* < 0.001; Figure S3). To further elucidate the relationship between antioxidant genes and aquatic microorganisms, correlation analysis was performed. All fungi showed negative correlations with antioxidant genes, while some bacteria (*Acinetobacter*, *Pseudomonas* and *Flavobacterium*) exhibited positive correlations with antioxidant genes (Table S1, Figure 5). Furthermore, the strength of the correlation between fungi and antioxidant genes was greater than that observed for bacteria. The microorganisms showing the strongest correlations with the superoxide dismutase‐encoding genes *sodA*, *sodB* and *sodC* were *Flavobacterium*, *Rozella* and *Acinetobacter*, respectively. The gene *gshA* (glutamate cysteine ligase) showed a strong positive correlation with *Pseudomonas* (*r* = 0.716, *p* < 0.001). Additionally, the gene *ahpC* showed a negative correlation with *Acidimicrobium* (*r* = −0.825, *p* < 0.001), whereas its correlation with *Flavobacterium* was positive (*r* = 0.817, *p* < 0.001). These results indicate that microorganisms expressing antioxidant genes exhibit an antagonistic relationship with fungi.

### 3.5 Correlation Analysis of the Nitrogen/Phosphorus (N/P) Cycling Genes and Planktonic Microorganisms

**FIGURE 6:**
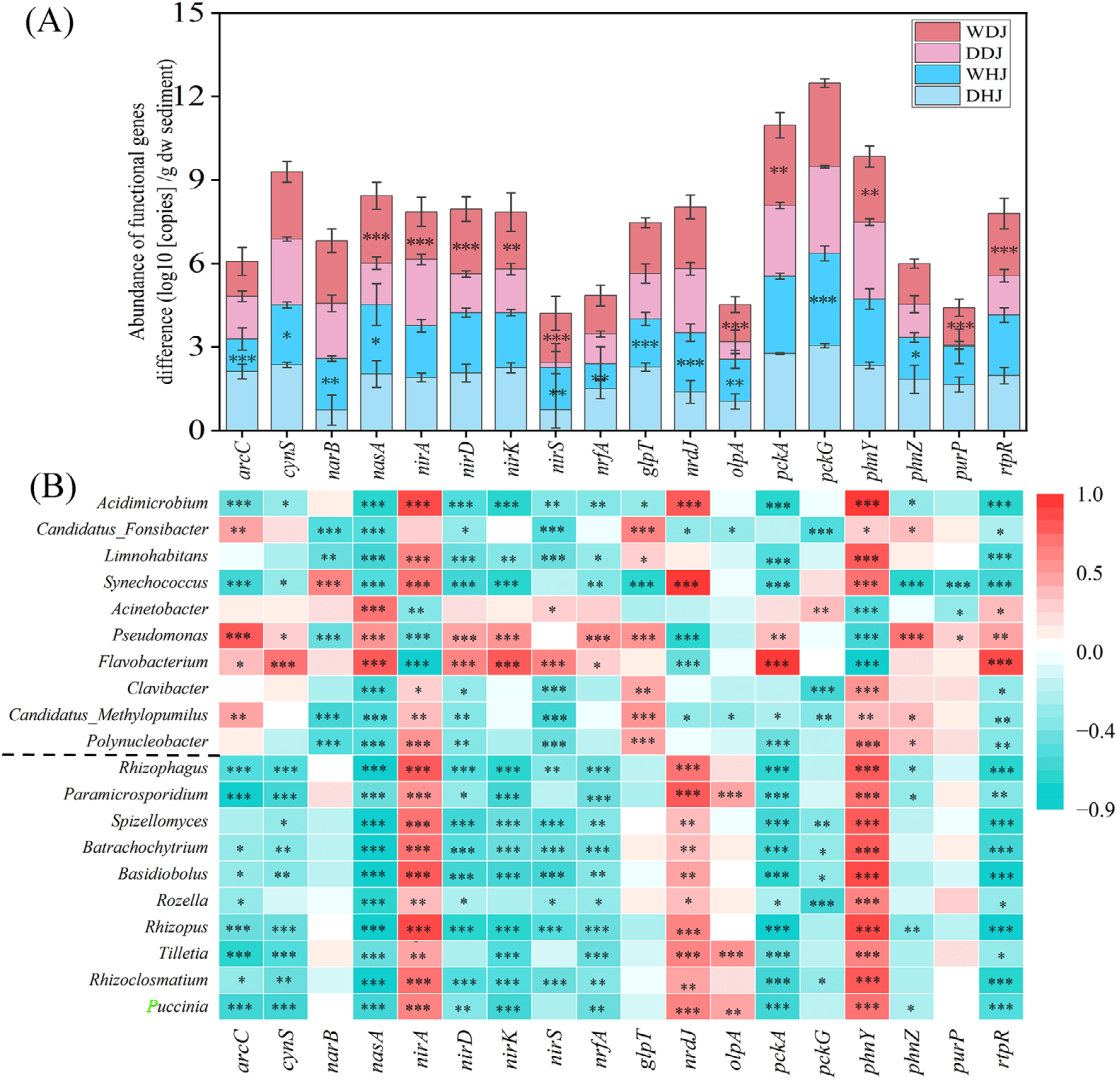
The abundance of N/P cycling genes and their correlation with microorganisms. (A) The abundance difference (log10 [copies]) of the N/P cycling genes. (B) Heat map of Pearson correlation analysis of the N/P cycling genes and bacterial (top 10) and fungal (bottom 10) at genus level. Asterisks above columns and close to numerical values denote significance (*p < 0.05; **p < 0.01; ***p < 0.001).

Compared to the dry season, the levels of most N/P cycling‐related genes showed significant changes during the wet season (Table S2, Figure 6A). Genes such as *nasA* (assimilatory nitrate reductase), *nirS* (nitrite reductase) and *olpA* (acid phosphatase) increased significantly in the wet season. Within the HJ, the abundance of genes *arcC* (carbamate kinase) and *glpT* (glycerol‐3‐phosphate transporter) significantly decreased during the wet season, while genes *nirA* (ferredoxin nitrite reductase) and *phnY* (phosphonoacetaldehyde dehydrogenase) exhibited significant decreases in the DJ. Furthermore, most genes showing significant changes in the WDJ displayed an increasing trend. Seasonal variation significantly altered the N/P cycling gene abundance, with observed differences between DJ and HJ.

Correlation analysis between N/P cycling genes and microorganisms revealed that these genes were predominantly negatively correlated with both bacteria and fungi (Table S2, Figure 6B). Gene *phnY* showed the highest positive correlation with *Acidimicrobium* (*r* = 0.96, *p* < 0.001), whereas gene *nasA* was strongly negatively correlated with *Basidiobolus* (*r* = −0.917, *p* < 0.001). The highest positive correlation for *nirA* was found with *Rhizopus* (*r* = 0.821, *p* < 0.001). Genes *nirA* and *phnY* were primarily positively correlated with microorganisms, except for *Acinetobacter*, *Pseudomonas* and *Flavobacterium*. However, genes *nasA* and *rtpR* (adenosylcobalamin‐dependent ribonucleoside‐triphosphate reductase) exhibited the opposite trend compared to genes *nirA* and *phnY*. Gene *nrdJ* (vitamin B12‐dependent ribonucleotide reductase) displayed a high positive correlation with *Synechococcus* (*r* = 0.924, *p* < 0.001) and a negative correlation with *Pseudomonas* (*r* = −0.756, *p* < 0.001).

### 3.6 Environmental Factors and Their Correlations With the Microbial Community

**FIGURE 7:**
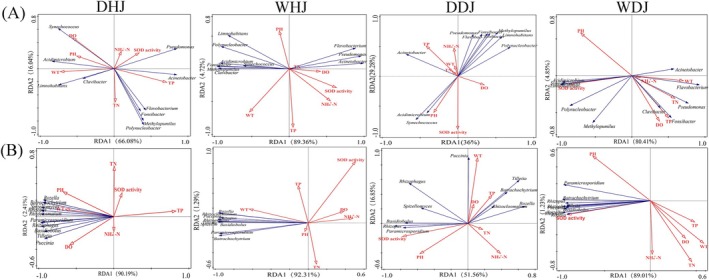
RDA biplot of microbial communities and physicochemical parameters. (A) Bacteria community. (B) Fungi community.

RDA was employed to evaluate how environmental factors in DJ and HJ influenced bacterial and fungal community variation. The TP and WT are the main influencing factors of DHJ and WHJ bacterial community structure, while pH and TP are the main influencing factors of DDJ and WDJ (Figure 7A). *Acidimicrobium* and *Synechococcus* showed positive correlations with pH across all samples, but negative correlations with TN and TP. *Acinetobacter* and *Pseudomonas* exhibited positive correlations with SOD enzyme activity in HJ but negative correlations in DJ, while *Synechococcus* and *Acidimicrobium* showed the opposite trend. Moreover, *Acinetobacter* showed a negative correlation with DO in the dry season but a positive correlation in the wet season.

The primary factors influencing DHJ, WHJ, DDJ and WDJ fungal community structure were pH and TP, WT and TN, WT and pH, and TN and pH, respectively (Figure 7B). *Rhizophagus* displayed negative correlations with TN across all samples but positive correlations with pH. *Paramicrosporidium* and *Rhizopus* were positively correlated with WT in HJ but negatively correlated in DJ. TP showed negative correlations with all fungi in DHJ but positive correlations in WHJ. *Paramicrosporidium*, *Rhizopus*, *Basidiobolus* and *Rhizophagus* exhibited a negative correlation with SOD activity in HJ, whereas a positive correlation was observed in DJ. Additionally, SOD activity showed positive correlations with all fungi in WDJ. In summary, the correlations between physicochemical properties and bacteria/fungi exhibited spatial heterogeneity across different seasons in DJ and HJ.

### 3.7 Microbial Community Assembly Patterns

**FIGURE 8:**
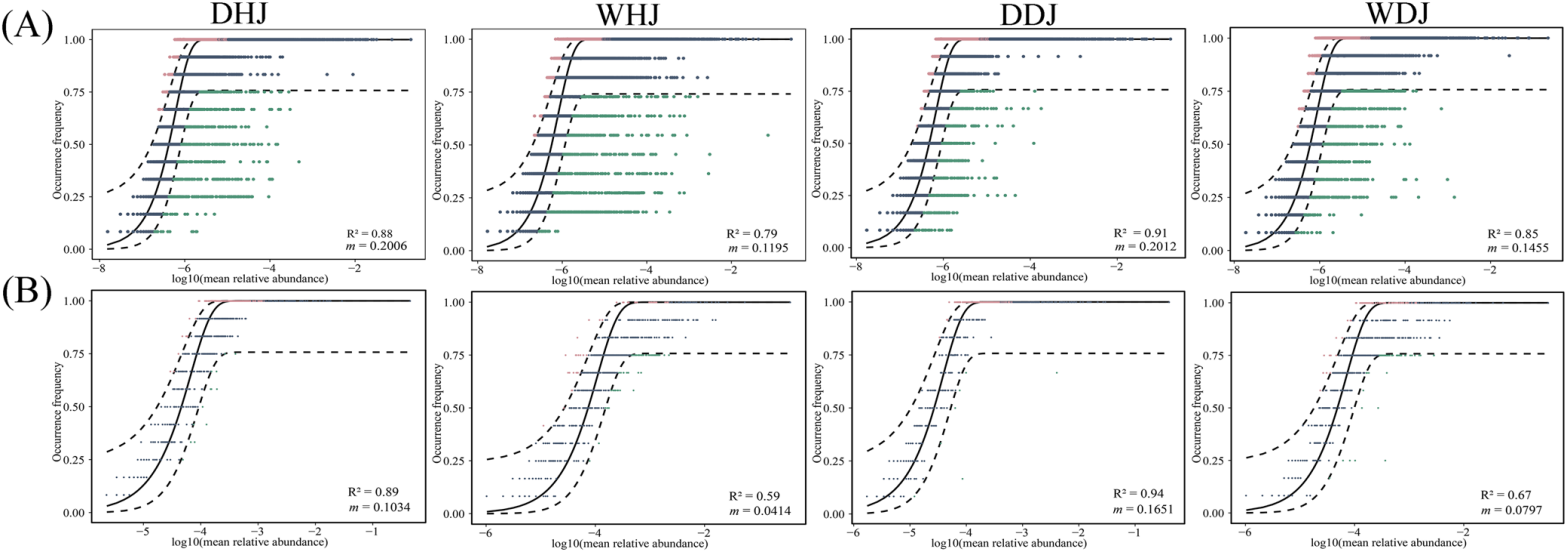
The neutral model analysis of microbial communities in DJKR. (A) Bacteria community. (B) Fungi community. The predicted occurrence frequency was shown as a solid blue line, while the dashed blue line marks the 95% confidence interval. The m indicates the immigration rate, while *R* ^2^ indicates the fit to the neutral model.

Using neutral community model analysis, the neutral model explained 87.85% of the variation for DHJ, 79.31% for WHJ, 91.10% for DDJ and 84.63% for WDJ in planktonic bacterial communities (Figure 8A). For fungal communities, it explained 88.65%, 59.17%, 93.99% and 67.43% of the variation, respectively (Figure 8B). Stochastic processes were found to be the primary drivers of bacterial and fungal community composition in HJ and DJ, with deterministic processes exerting less influence. Furthermore, a more pronounced effect of stochastic processes on microbial communities was observed during the dry season than the wet season. Both bacterial and fungal communities exhibited higher migration rates in the dry season than in the wet season, indicating that microbial communities during the wet season are more limited by dispersal. Across different periods, fungal communities had a lower niche breadth than bacterial communities, indicating greater constraints on fungal communities in HJ and DJ.

## 4 Discussion

The DJKR is the primary water source for the Middle Route of the South‐to‐North Water Diversion Project, located on the border of Henan and Hubei provinces in China (Webber et al. 2017). Nevertheless, the DJKR microbial plankton community exhibits disturbances attributable to water transfer activities (Chen et al. 2018; Yang et al. 2023). High‐throughput sequencing was employed in this study to examine the composition and diversity of microbial communities. In comparison with the dry season, planktonic bacterial diversity showed a decrease in HJ but an increase in DJ (Figure 2). It has been found that the diversity of bacterioplankton in DJ was lower during the wet season compared to the dry season (Yang et al. 2023), possibly due to differences in sampling points and time. The variability of fungal community composition in HJ and DJ during the wet season was lower than that during the dry season, indicating that seasonal hydrological changes promote the homogenization of community structure. This phenomenon coincided with concurrent declines in TN and NH_4_^+^‐N concentrations in wet season, suggesting that eutrophication alleviation may be a key driving factor. Eutrophication may lead to community homogenization, thereby exacerbating the risk of biodiversity loss (Geng et al. 2022). While this study validates the above pattern, the interactive mechanisms between eutrophication and fungal community structure require further elucidation.

In agreement with earlier reports, the dominant phyla in the bacterioplankton communities of all samples (DHJ, WHJ, DDJ and WDJ) were Proteobacteria and Actinobacteria (Chen, Liu, et al. 2022). In DJ, a decrease in Proteobacteria relative abundance was observed during the wet season, accompanied by an increase in Actinobacteria (Figure 3A). The same trend was also observed in Poyang Lake (Ren et al. 2019). β‐proteobacteria, a class within the Proteobacteria phylum, are known for nitrogen fixation and are predominantly chemoorganotrophs. Moreover, Proteobacteria as a whole are closely associated with water pollution and can serve as reliable indicators of heavy metal contamination levels (Sui et al. 2020; Xu et al. 2023). Actinobacteria were the primary contributors to off‐flavours during non‐algal bloom periods, and their secondary metabolites include semi‐volatile terpenoids, the production of which is influenced by rainfall (Asquith et al. 2018; Zuo et al. 2010). The dominant genera were *Acidimicrobium* and *Candidatus_Fonsibacter*. And *Acidimicrobium* can degrade organic pollutants and has certain potential in bioremediation (Jaffé et al. 2024). The dominant genera in Sancha Lake were *Pseudomonas* and *Bacillus* (Li, Gong, et al. 2025), whereas *Comamonadaceae_unclassified* and *Flavobacterium* were dominant genus in the Han River (Sun et al. 2021). The composition of bacterial genera varied across various aquatic ecosystems, indicating that heterogeneity in environmental factors driven by spatiotemporal variations has a significant impact on community structure at the genus level.

Among known fungal phyla, Basidiomycota and Ascomycota predominated among the planktonic fungal communities in all samples; the community composition was similar to the 5 sites from DJ and Songhua river in Northeast China (Chen et al. 2018; Li, Zhao, et al. 2023). High‐throughput sequencing technology has led to significant progress in the study of aquatic microorganisms, but compared to fungal communities in terrestrial environments, freshwater fungal communities remain poorly understood (Sui et al. 2020). Notably, a significant portion of fungal taxa have not yet been identified at higher classification levels. Our metagenomic analysis revealed that the planktonic fungi in DJ and HJ comprised 9 phyla and 465 genera, surpassing the 7 phyla and 294 genera detected in DJ using 18S sequencing (Chen et al. 2018). This indicates that HJ also has a relatively rich planktonic fungal community. Basidiomycota drive carbon and nitrogen cycling by decomposing organic matter in river sediments, processing complex organics into inorganic end products (Ghobad‐Nejhad et al. 2023). Basidiomycota abundance showed a higher value in the wet season than in the dry season, which may be due to rainwater washing plant residues into the DJKR.

Metabolism, which is essential for organisms, shows the highest relative abundance in KEGG Level 1. Seasonal changes significantly affect bacterial metabolic functions and also strongly influence fungal functions, including environmental information processing, cellular processes and genetic information processing (Figure 4). During the dry season, reduced rainfall, lower biological consumption and enhanced nutrient release from sediments collectively lead to higher nutrient concentrations and poorer water quality compared to the wet season (Hideo et al. 2017; Li et al. 2016). Such seasonal nutrient dynamics may lead to seasonal differences in the functions of bacterial and fungal communities. Bacterioplankton primarily participated in decomposing organic matter and mineralizing organic compounds into inorganic nutrients, a process that is crucial for nutrient cycling (Geng et al. 2022). Bacterioplankton in aquatic environments exhibit heightened sensitivity to external changes due to their direct exposure in water. This enables them to respond more readily to alterations in physicochemical parameters and human‐made pollutants (Worsfold et al. 2008). And fungal communities primarily contribute to material cycling through their roles as parasites/pathogens or symbionts for higher organisms (Siriarchawatana et al. 2024). This suggested that the sensitivity of bacteria might be stronger than that of fungi, which was consistent with the higher number of significantly different metabolic pathways observed in bacteria in this study (Figure 4).

In KEGG annotation Level 2, amino acid metabolism and carbohydrate metabolism were identified as the dominant pathways in both bacteria and fungi, with an increase in their relative abundance being seen during the wet season. These two metabolic pathways were also the most active functions in Chishui river (China) sediment (Di et al. 2024). Amino acids are the basic structural units of protein synthesis and can also regulate intracellular pH and ion transport to maintain normal life activities, and amino acid metabolism has a close relationship with the nitrogen cycle (Zhang et al. 2012; Huang 2019). Furthermore, carbohydrate metabolism abundance was > 9% in bacteria and > 6% in fungi, which abundance only significantly increased in bacteria (*p* < 0.001). Carbohydrates are essential for nutrient cycling, and carbohydrate‐active enzymes are used by microorganisms to decompose polysaccharides and obtain energy (Li, Cheng, et al. 2024). Furthermore, carbohydrates serve as fundamental structural components of microbial cells. Enhanced metabolic capacity facilitates carbohydrate biosynthesis, thereby promoting microbial degradation of environmental contaminants (Zhang et al. 2021). Additionally, compared with the dry season, bacterial lipid metabolism was significantly enhanced under wet season, whereas fungal communities exhibited a marked reduction in this function under wet season (Figure 4). It has been documented in previous studies that, in a seasonally hypoxic estuary, planktonic bacterial richness was strongly negatively associated with DO (Spietz et al. 2015), whereas a significant positive correlation was observed between DO and planktonic fungal richness in DJKR (Chen et al. 2018). Therefore, the wet‐season DO decrease in HJ and DJ (Figure 1) likely promoted bacterial abundance and lipid metabolism but reduced fungal abundance and lipid metabolism. The above findings collectively demonstrate that both bacteria and fungi can resist environmental perturbations by selectively modulating their metabolic pathways, with bacteria exhibiting greater sensitivity to seasonal changes than fungi.

The presence of pollutants (e.g., heavy metals or microplastics) in water bodies induces oxidative stress in planktonic microorganisms, subsequently leading to the generation of reactive oxygen species (ROS) (Chen, Wu, et al. 2022; Jasińska et al. 2022). Antioxidants can eliminate ROS and prevent it from causing damage to microorganisms (Genç et al. 2020). Antioxidants mainly include superoxide dismutase, catalase, glutathione peroxidase, and so on. In this study, the abundance of most antioxidant‐related genes did not exhibit significant changes under both seasons (dry and wet) (Figure S3). However, SOD activity in both DJ and HJ was higher in the wet season than in the dry season (Figure 1). This might be explained by a temporal discrepancy between gene expression and protein translation. Correlation analysis showed that antioxidant genes exhibited a negative correlation with planktonic fungi, whereas a positive correlation was observed only with some bacteria, specifically *Acinetobacter*, *Pseudomonas* and *Flavobacterium* (Figure 5). *Acinetobacter* can efficiently degrades a wide range of compounds (Wang, Wang, Wang, et al. 2025). *Pseudomonas* is ubiquitous microorganisms capable of degrading various types of pollutants, such as metals (chromium and cadmium), hydrocarbons, phenol and pesticides (Kotresha and Vidyasagar 2017; Hassen and Abbassi 2025). *Flavobacterium* can convert organic nitrogen into ammonium nitrogen, significantly promoting nitrogen cycling in water bodies (Li, Wang, et al. 2024). RDA analysis revealed a positive correlation between SOD activity and *Acinetobacter*, *Pseudomonas* and *Flavobacterium* in the HJ, whereas a negative correlation with these three genera was observed in the DJ. The higher SOD activity coupled with lower levels of TN and TP in the DJ jointly suggest that these bacterial genera may be linked to water quality improvement. The influence of SOD activity on fungal communities differs moderately across different sites. The colonization of *Rhizophagus irregularis* in the roots of cotton can increase the SOD activity of the roots, enhancing their drought resistance (Luo et al. 2024). However, few investigations currently have been conducted on the oxidative stress of planktonic fungi.

In the study, regional and seasonal factors have varying degrees of impact on N/P cycle‐related genes abundance. The N/P cycle‐related genes were mainly negatively correlated with bacteria and fungi (Figure 6). *Acidimicrobium* exhibited a high positive correlation with both *nirA* and *phny* (Table S2). The *Acidimicrobium* was commonly found in iron‐rich, acidic sediments, which can perform anaerobic ammonium oxidation under iron‐reducing conditions as well as polyfluoroalkyl substances bioremediation (Huang et al. 2024). *Flavobacterium* can also participate in ammonification in the nitrogen cycle (Li, Wang, et al. 2024), except for the gene *nirA*, which was positively correlated with other nitrogen cycle genes. However, gene *nirA* also encodes ferredoxin‐nitrite reductase, which reduces nitrate to ammonia (Fenn et al. 2021). A positive correlation exists between *Pseudomonas* and the gene *nirA* (*r* = −0.486, *p* < 0.001), the nitrite reductase NirA can act as a promising antivirulence target candidate against *Pseudomonas aeruginosa* infections (Fenn et al. 2021). *Synechococcus* can serve as a biological indicator of eutrophication in aquatic ecosystems, which has a high positive correlation with gene *nrdJ*. Ribonucleotides are reduced to their corresponding deoxyribonucleotides by the NrdJ protein, which has been found to be mainly involved in DNA repair, and potentially in DNA replication when oxygen levels are low in *Pseudomonas aeruginosa* (Torrents et al. 2005). Eutrophication in water bodies can lead to a decrease in DO and trigger blooms of *Synechococcus*, which require the NrdJ protein for reproduction. *Rhizophagus* was significantly positively correlated with the gene *nirA* and *phnY*. *Rhizophagus* generally coexist with plant rhizosphere, and their hyphae can provide nitrogen and phosphorus to plants, while plants provide more carbon for fungal growth and metabolism (Wang et al. 2020; Andrino et al. 2020). The presence of *Rhizophagus* may be due to the fact that plant root residues enter the DJKR with the river.

In the present study, different environmental factors in different seasons and sampling locations have varying impacts on the planktonic microbial community. At genera level, RDA indicated that both bacterial and fungal community structures were associated with pH, WT, TP and TN. Aerobic microorganisms require oxygen for growth and development, and dissolved oxygen also serves as a major determinant of planktonic microbial community structure (Wu et al. 2021). The level of DO decreases during the wet season in both DJ and HJ (Figure 1). And some partial aerobic bacteria (*Acidemebium*, *Limnohabitans* and *Synechococcus*) and all fungi have negatively correlated with DO during the wet season. Physicochemical parameters (e.g., pH, TN, WT and TP) also shaped the structure and composition of microbial communities in water (Liu et al. 2020; Li, Chen, et al. 2025). Seasonal variation was also found to significantly modify both bacterial community composition and water quality (Yan et al. 2017; Wang, Liu, et al. 2023). Microorganisms are the key drivers of nitrogen and phosphorus cycles in aquatic ecosystems. Previous studies on the bacterial community in DJKR have identified N and P nutrients as the main drivers of the community (Chen, Liu, et al. 2022). And the distribution of planktonic fungi was strongly influenced by DO, TN and chlorophyll a (Chen et al. 2018). The correlations between TN, TP and microorganisms were found in our study to vary with location and season.

The study of how microbial communities assemble currently stands at the forefront of microbial ecology (Li et al. 2021). Neutral models together with standardized random rates enable robust quantification of deterministic and stochastic contributions to community assembly (Luo et al. 2019). Research found that both dispersal limitation and environmental selection play a role in influencing how microbial communities assemble (Huang et al. 2023). The shifts in HJ and DJ microbial communities in this study were mainly influenced by stochastic processes. A good fit of the neutral community model was observed for the bacterial and fungal communities during the dry season (*R* ^2^ = 0.8785 ~ 0.9399), while only a moderate fit was obtained in the wet season (*R* ^2^ = 0.5917 ~ 0.8463). These findings point to stochastic processes as the main drivers of community aggregation in this study, but the community assembly in DJKR at different water depths and years only played a moderate role (Chen, Liu, et al. 2022; Wang, Wang, Li, et al. 2025). Water storage and transfer demands influence the water quality of DJKR. As a result, its physicochemical properties exhibit strong seasonal variations annually. This dynamic likely disrupts neutral processes, leading to the predominance of stochastic processes. Comparing the migration rates (*m*) of different microbial communities reflects differences in their dispersal capabilities. In this study, bacteria showed a higher migration rate than fungi in DJKR, implying that the fungal community might be more susceptible to dispersal limitation. This difference might be attributed to the typically greater environmental adaptability and broader dispersal mechanisms of bacteria (Chen, Wang, et al. 2020). The *m* of microbial communities decreased in the wet season relative to the dry season. This difference probably resulted from physicochemical properties changes in the water, which restricted microbial dispersal.

## 5 Conclusions

A comprehensive analysis was conducted on the bacterial and fungal communities in the DJKR, aiming to clarify how seasonal changes shape microbial distribution and community assembly. The DJKR harboured more abundant bacterial than fungal communities, but bacterial diversity was lower. Fungal diversity also declined in the wet season relative to the dry season. Bacteria were dominated by Proteobacteria and Actinobacteria, while fungi were dominated by Basidiomycota and Ascomycota. WT, pH and TP strongly determine the genus‐level community structure of bacterial and fungal communities. The assembly process of planktonic bacteria and fungi in HJ and DJ was mainly influenced by stochastic processes. Compared with the dry season, deterministic processes exert a stronger influence on the DJKR in the wet season. This study offers insights into environmental impacts on planktonic microbial communities and lays a foundation for using them as indicators of reservoir water quality and water source ecological functions.

## Author Contributions

**Yarui Cheng:** investigation, writing – original draft, writing – review and editing, formal analysis, software, project administration. **Lu Gan:** formal analysis, data curation. **Yaqing Zhou:** data curation, investigation. **Fengjian Wang:** funding acquisition, methodology, resources. **Xiaoyu Lu:** data curation, validation. **Zunwei Ke:** resources, data curation, methodology.

## Funding

This work was supported by the National Natural Science Foundation of China (32400051), Program for Science and Technology Innovation Team in Colleges of Hubei Province (T201928) and Scientific Research Project of Hanjiang Normal University (XJ2022A02).

## Conflicts of Interest

The authors declare no conflicts of interest.

## Supporting information


**Figure S1:** Distribution of sampling sites in the DJKR: a total of 8 sampling sites (4 sampling sites at HJ and DJ respectively).

**Figure S2:** The relative abundances of global and overview maps. Different lowercase letters indicate the difference is significant (*p* < 0.05).

**Figure S3:** The abundance difference (log10 [copies]) of the antioxidant genes. Asterisks above columns and close to numerical values denote significance: wet season vs. dry season (*p < 0.05; **p < 0.01; ***p < 0.001).

**Table S1:** The data of antioxidant genes and microorganisms abundance and correlation calculation. Sheet 1: The abundance of the antioxidant genes. Sheet 2: The abundance of bacterial (top 10) and fungal (bottom 10) at genus level. Sheet 3: Correlation and significance calculation of microbial and antioxidant genes.

**Table S2:** The data of nitrogen/phosphorus cycling genes abundance and correlation calculation with microorganism. Sheet 1: The abundance of the nitrogen/phosphorus cycling genes. Sheet 2: Correlation and significance calculation of microbial and nitrogen/phosphorus cycling genes.

## Data Availability

The data that support the findings of this study are openly available in National Center for Biotechnology Information at https://www.ncbi.nlm.nih.gov/, reference number PRJNA1320825.

## References

[emi470362-bib-0001] Andrino, A. , G. Guggenberger , L. Sauheitl , S. Burkart , and J. Boy . 2020. “Carbon Investment Into Mobilization of Mineral and Organic Phosphorus by Arbuscular Mycorrhiza.” Biology and Fertility of Soils 57: 47–64. 10.1007/s00374-020-01505-5.

[emi470362-bib-0002] Asquith, E. , C. Evans , R. Dunstan , P. Geary , and B. Cole . 2018. “Distribution, Abundance and Activity of Geosmin and 2‐Methylisoborneol‐Producing Streptomyces in Drinking Water Reservoirs.” Water Research 145: 30–38. 10.1016/j.watres.2018.08.014. 30118975

[emi470362-bib-0003] Chen, J. , P. Wang , C. Wang , et al. 2020. “Fungal Community Demonstrates Stronger Dispersal Limitation and Less Network Connectivity Than Bacterial Community in Sediments Along a Large River.” Environmental Microbiology 22, no. 3: 832–849. 10.1111/1462-2920. 31469494

[emi470362-bib-0004] Chen, K. , X. Wu , Z. Zou , et al. 2022. “Assess Heavy Metals‐Induced Oxidative Stress of Microalgae by Electro‐Raman Combined Technique.” Analytica Chimica Acta 1208: 339791. 10.1016/j.aca.2022.339791. 35525583

[emi470362-bib-0005] Chen, W. , K. Ren , A. Isabwe , H. Chen , M. Liu , and J. Yang . 2019. “Stochastic Processes Shape Microeukaryotic Community Assembly in a Subtropical River Across Wet and Dry Seasons.” Microbiome 7, no. 1: 138. 10.1186/s40168-019-0749-8. 31640783 PMC6806580

[emi470362-bib-0006] Chen, Z. , Y. Liu , Y. Li , et al. 2022. “The Seasonal Patterns, Ecological Function and Assembly Processes of Bacterioplankton Communities in the Danjiangkou Reservoir, China.” Frontiers in Microbiology 13: 884765. 10.3389/fmicb.2022.884765. 35783417 PMC9240478

[emi470362-bib-0007] Chen, Z. , G. Xu , C. Ding , et al. 2020. “Illumina MiSeq Sequencing and Network Analysis the Distribution and Co‐Occurrence of Bacterioplankton in Danjiangkou Reservoir, China.” Archives of Microbiology 202: 859–873. 10.1007/s00203-019-01798-7. 31894394

[emi470362-bib-0008] Chen, Z. , J. Yuan , F. Sun , et al. 2018. “Planktonic Fungal Community Structures and Their Relationship to Water Quality in the Danjiangkou Reservoir, China.” Scientific Reports 8, no. 1: 10596. 10.1038/s41598-018-28903-y. 30006549 PMC6045663

[emi470362-bib-0009] Di, F. , D. Han , G. Wang , et al. 2024. “Characteristics of Bacterial Community Structure in the Sediment of Chishui River (China) and the Response to Environmental Factors.” Journal of Contaminant Hydrology 263: 104335. 10.1016/j.jconhyd.2024.104335. 38520935

[emi470362-bib-0010] Dini‐Andreote, F. , J. C. Stegen , J. D. van Elsas , and J. F. Salles . 2015. “Disentangling Mechanisms That Mediate the Balance Between Stochastic and Deterministic Processes in Microbial Succession.” Proceedings of the National Academy of Sciences of the United States of America 112, no. 11: E1326–E1332. 10.1073/pnas.1414261112. 25733885 PMC4371938

[emi470362-bib-0011] Fang, K. , G. Xu , X. Chen , J. Li , Y. Cheng , and Y. Cheng . 2025. “Distribution Pattern and Assembly Process of Fungal Communities Along Altitude Gradient in Sediments of the Yellow River Basin.” Journal of Fungi (Basel, Switzerland) 11, no. 3: 214. 10.3390/jof11030214. 40137252 PMC11943069

[emi470362-bib-0012] Fenn, S. , J. F. Dubern , C. Cigana , et al. 2021. “NirA Is an Alternative Nitrite Reductase From *Pseudomonas aeruginosa* With Potential as an Antivirulence Target.” MBio 12, no. 2: e00207‐21. 10.1128/mBio.00207-21. 33879591 PMC8092218

[emi470362-bib-0013] Genç, Y. , H. Bardakci , Ç. Yücel , et al. 2020. “Oxidative Stress and Marine Carotenoids: Application by Using Nanoformulations.” Marine Drugs 18, no. 8: 423. 10.3390/md18080423. 32823595 PMC7459739

[emi470362-bib-0014] Geng, M. , W. Zhang , T. Hu , R. Wang , X. Cheng , and J. Wang . 2022. “Eutrophication Causes Microbial Community Homogenization via Modulating Generalist Species.” Water Research 210: 118003. 10.1016/j.watres.2021.118003. 34982976

[emi470362-bib-0015] Ghobad‐Nejhad, M. , B. Dima , B. K. Cui , and J. Si . 2023. “Editorial: Basidiomycete Fungi: From Biosystematics and Biodiversity to Biotechnology.” Frontiers in Microbiology 14: 1128319. 10.3389/fmicb.2023.1128319. 36778884 PMC9910330

[emi470362-bib-0016] Gurevich, A. , V. Saveliev , N. Vyahhi , and G. Tesler . 2013. “QUAST: Quality Assessment Tool for Genome Assemblies.” Bioinformatics 29, no. 8: 1072–1075. 10.1093/bioinformatics/btt086. 23422339 PMC3624806

[emi470362-bib-0017] Hao, Z. , Y. Wang , E. Chen , et al. 2025. “Climate and Biological Factors Jointly Shape Microbial Community Structure in the Yarlung Zangbo River During the Dry Season.” Science of the Total Environment 969: 178930. 10.1016/j.scitotenv.2025.178930. 40020580

[emi470362-bib-0018] Hassen, B. , and M. S. Abbassi . 2025. “Molecular Mechanisms of Heavy Metal Resistance and Cross−/Co‐Resistance to Antibiotics in *Pseudomonas aeruginosa* .” Letters in Applied Microbiology 78, no. 7: ovaf094. 10.1093/lambio/ovaf094. 40650566

[emi470362-bib-0019] Hideo, O. , E. Shuichi , I. Toshiyuki , O. Yasuaki , and T. Shinji . 2017. “Seasonal Changes in Water Quality as Affected by Water Level Fluctuations in Lake Tonle Sap, Cambodia.” Geographical Review of Japan Series B 90, no. 2: 53–65. 10.4157/geogrevjapanb.90.53.

[emi470362-bib-0020] Hu, A. , F. Ju , L. Hou , et al. 2017. “Strong Impact of Anthropogenic Contamination on the Co‐Occurrence Patterns of a Riverine Microbial Community.” Environmental Microbiology 19, no. 12: 4993–5009. 10.1111/1462-2920.13942. 28967165

[emi470362-bib-0021] Huang, Y. 2019. “Illumina‐Based Analysis of Endophytic Bacterial Diversity of Four Allium Species.” Scientific Reports 9, no. 1: 15271. 10.1038/s41598-019-51707-7. 31649302 PMC6813343

[emi470362-bib-0022] Huang, S. , C. Smorada , C. E. Schaefer , and P. R. Jaffé . 2024. “Stimulating Acidimicrobium sp. Strain A6 in Iron‐Rich, Acidic Sediments From AFFF‐Impacted Sites for PFAS Defluorination.” Science of the Total Environment 955: 176801. 10.1016/j.scitotenv.2024.176801. 39389130

[emi470362-bib-0023] Huang, Z. , B. Pan , X. Zhao , X. Liu , X. Liu , and G. Zhao . 2023. “Hydrological Disturbances Enhance Stochastic Assembly Processes and Decrease Network Stability of Algae Communities in a Highland Floodplain System.” Science of the Total Environment 903: 166207. 10.1016/j.scitotenv.2023.166207. 37567295

[emi470362-bib-0024] Jaffé, P. R. , S. Huang , J. Park , M. Ruiz‐Urigüen , W. Shuai , and M. Sima . 2024. “Defluorination of PFAS by Acidimicrobium sp. Strain A6 and Potential Applications for Remediation.” Methods in Enzymology 696: 287–320. 10.1016/bs.mie.2024.01.013. 38658084 PMC11895404

[emi470362-bib-0025] Jasińska, A. , S. Różalska , V. Rusetskaya , M. Słaba , and P. Bernat . 2022. “Microplastic‐Induced Oxidative Stress in Metolachlor‐Degrading Filamentous Fungus Trichoderma Harzianum.” International Journal of Molecular Sciences 23, no. 21: 12978. 10.3390/ijms232112978. 36361770 PMC9658726

[emi470362-bib-0026] Keneally, C. , D. Chilton , T. N. Dornan , et al. 2025. “Multi‐Omics Reveal Microbial Succession and Metabolomic Adaptations to Flood in a Hypersaline Coastal Lagoon.” Water Research 280: 123511. 10.1016/j.watres.2025.123511. 40147302

[emi470362-bib-0027] Kotresha, D. , and G. M. Vidyasagar . 2017. “Phenol Degradation in a Packed Bed Reactor by Immobilized Cells of *Pseudomonas aeruginosa* MTCC 4997.” Biocatalysis and Agricultural Biotechnology 10: 386–389. 10.1016/j.bcab.2017.04.015.

[emi470362-bib-0028] Li, B. , G. Yang , R. Wan , Y. Zhang , X. Dai , and Y. Chen . 2016. “Spatiotemporal Variability in the Water Quality of Poyang Lake and Its Associated Responses to Hydrological Conditions.” Water 8: 296. 10.3390/w8070296.

[emi470362-bib-0029] Li, C. , L. Jin , C. Zhang , et al. 2023. “Destabilized Microbial Networks With Distinct Performances of Abundant and Rare Biospheres in Maintaining Networks Under Increasing Salinity Stress.” iMeta 2, no. 1: e79. 10.1002/imt2.79. 38868331 PMC10989821

[emi470362-bib-0030] Li, D. , C. Liu , R. Luo , K. Sadakane , and T. Lam . 2015. “MEGAHIT: An Ultra‐Fast Single‐Node Solution for Large and Complex Metagenomics Assembly via Succinct de Bruijn Graph.” Bioinformatics 31, no. 10: 1674–1676. 10.1093/bioinformatics/btv033. 25609793

[emi470362-bib-0031] Li, M. , T. Mi , H. He , Y. Chen , Y. Zhen , and Z. Yu . 2021. “Active Bacterial and Archaeal Communities in Coastal Sediments: Biogeography Pattern, Assembly Process and Co‐Occurrence Relationship.” Science of the Total Environment 750: 142252. 10.1016/j.scitotenv.2020.142252. 33182220

[emi470362-bib-0032] Li, M. , T. Zhao , D. Liang , et al. 2023. “Diversity Characterization of Bacteria and Fungi in Water, Sediments and Biofilms From Songhua River in Northeast China.” Chemosphere 338: 139524. 10.1016/j.chemosphere.2023.139524. 37467849

[emi470362-bib-0033] Li, X. , X. Cheng , J. Xu , et al. 2024. “Dynamic Patterns of Carbohydrate Metabolism Genes in Bacterioplankton During Marine Algal Blooms.” Microbiological Research 286: 127785. 10.1016/j.micres.2024.127785. 38851011

[emi470362-bib-0034] Li, Y. , W. Chen , Y. Han , et al. 2025. “Taxon‐Dependent Community Assembly of Bacteria and Protists in River Ecosystems: A Case Study From the Yujiang River.” Microorganisms 13, no. 7: 1650. 10.3390/microorganisms13071650. 40732159 PMC12298076

[emi470362-bib-0035] Li, Y. , S. Gong , H. Liu , Y. Li , W. Luo , and Z. Gong . 2025. “Bacterioplankton Community Structure and Molecular Ecological Network Characteristics in the Overlying Water of Sancha Lake.” PLoS One 20, no. 7: e0327903. 10.1371/journal.pone.0327903. 40663539

[emi470362-bib-0036] Li, Y. , X. Wang , T. Deng , et al. 2024. “ *Flavobacterium poyangense* sp. Nov., an Ammonifying Bacterium Isolated From a Freshwater Lake.” International Journal of Systematic and Evolutionary Microbiology 74, no. 6: 006416. 10.1099/ijsem.0.006416. 38865183

[emi470362-bib-0037] Liu, H. , J. Yin , and L. Feng . 2018. “The Dynamic Changes in the Storage of the Danjiangkou Reservoir and the Influence of the South‐North Water Transfer Project.” Scientific Reports 8, no. 1: 8710. 10.1038/s41598-018-26788-5. 29880825 PMC5992209

[emi470362-bib-0038] Liu, Z. , M. Iqbal , Z. Zeng , et al. 2020. “Comparative Analysis of Microbial Community Structure in the Ponds With Different Aquaculture Model and Fish by High‐Throughput Sequencing.” Microbial Pathogenesis 142: 104101. 10.1016/j.micpath.2020.104101. 32109568

[emi470362-bib-0039] Luo, C. , Z. Li , Y. Shi , et al. 2024. “Arbuscular Mycorrhizal Fungi Enhance Drought Resistance in *Bombax ceiba* by Regulating SOD Family Genes.” PeerJ 12: e17849. 10.7717/peerj.17849. 39131625 PMC11316461

[emi470362-bib-0040] Luo, Z. , J. Liu , P. Zhao , T. Jia , C. Li , and B. Chai . 2019. “Biogeographic Patterns and Assembly Mechanisms of Bacterial Communities Differ Between Habitat Generalists and Specialists Across Elevational Gradients.” Frontiers in Microbiology 10: 169. 10.3389/fmicb.2019.00169. 30804920 PMC6378303

[emi470362-bib-0041] Mohapatra, M. , P. Behera , J. Y. Kim , and G. Rastogi . 2020. “Seasonal and Spatial Dynamics of Bacterioplankton Communities in a Brackish Water Coastal Lagoon.” Science of the Total Environment 705: 134729. 10.1016/j.scitotenv.2019.134729. 31838414

[emi470362-bib-0042] Pinhassi, J. , H. Farnelid , S. M. García , et al. 2022. “Functional Responses of Key Marine Bacteria to Environmental Change—Toward Genetic Counselling for Coastal Waters.” Frontiers in Microbiology 13: 869093. 10.3389/fmicb.2022.869093. 36532459 PMC9751014

[emi470362-bib-0043] Ren, Z. , X. Qu , M. Zhang , Y. Yu , and W. Peng . 2019. “Distinct Bacterial Communities in Wet and Dry Seasons During a Seasonal Water Level Fluctuation in the Largest Freshwater Lake (Poyang Lake) in China.” Frontiers in Microbiology 10: 1167. 10.3389/fmicb.2019.01167. 31164883 PMC6536640

[emi470362-bib-0044] Siriarchawatana, P. , P. Harnpicharnchai , C. Phithakrotchanakoon , et al. 2024. “Fungal Communities as Dual Indicators of River Biodiversity and Water Quality Assessment.” Water Research 253: 121252. 10.1016/j.watres.2024.121252. 38340699

[emi470362-bib-0045] Spietz, R. L. , C. M. Williams , G. Rocap , and M. C. Horner‐Devine . 2015. “A Dissolved Oxygen Threshold for Shifts in Bacterial Community Structure in a Seasonally Hypoxic Estuary.” PLoS One 10, no. 8: e0135731. 10.1371/journal.pone.0135731. 26270047 PMC4535773

[emi470362-bib-0046] Sui, H. , J. Wang , Z. Li , et al. 2020. “Screening of Ecological Impact Assessment Indicators in Urban Water Body Restoration Process Itle.” Ecological Indicators 113: 106198. 10.1016/j.ecolind.2020.106198.

[emi470362-bib-0047] Sun, H. , B. Pan , H. He , et al. 2021. “Characterization of the Bacterioplankton Community and the Influencing Factors in the Upper Reaches of the Han River Basin.” Environmental Science and Pollution Research International 28, no. 43: 61748–61759. 10.1007/s11356-021-14906-2. 34189692

[emi470362-bib-0048] Torrents, E. , A. Poplawski , and B. M. Sjöberg . 2005. “Two Proteins Mediate Class II Ribonucleotide Reductase Activity in *Pseudomonas aeruginosa* : Expression and Transcriptional Analysis of the Aerobic Enzymes.” Journal of Biological Chemistry 280, no. 17: 16571–16578. 10.1074/jbc.M501322200. 15722359

[emi470362-bib-0049] Wang, C. , Y. Mao , W. Zhou , et al. 2023. “Inhomogeneous Antibiotic Distribution in Sediment Profiles in Anthropogenically Impacted Lakes: Source Apportionment, Fate Drivers, and Risk Assessment.” Journal of Environmental Management 341: 118048. 10.1016/j.jenvman.2023.118048. 37141721

[emi470362-bib-0050] Wang, H. , X. Liu , Y. Wang , et al. 2023. “Spatial and Temporal Dynamics of Microbial Community Composition and Factors Influencing the Surface Water and Sediments of Urban Rivers.” Journal of Environmental Sciences (China) 124: 187–197. 10.1016/j.jes.2021.10.016. 36182129

[emi470362-bib-0051] Wang, R. , J. Wang , L. Wang , et al. 2025. “A Novel Eco‐Friendly Acinetobacter Strain A1‐4‐2 for Bioremediation of Aquatic Pollutants.” Scientific Reports 15, no. 1: 23207. 10.1038/s41598-025-05431-0. 40603380 PMC12222735

[emi470362-bib-0052] Wang, S. , A. Chen , K. Xie , et al. 2020. “Functional Analysis of the OsNPF4.5 Nitrate Transporter Reveals a Conserved Mycorrhizal Pathway of Nitrogen Acquisition in Plants.” Proceedings of the National Academy of Sciences of the United States of America 117, no. 28: 16649–16659. 10.1073/pnas.2000926117. 32586957 PMC7368293

[emi470362-bib-0053] Wang, W. , R. Wang , Y. Li , et al. 2025. “Cross‐Sectional‐Dependent Microbial Assembly and Network Stability: Bacteria Sensitivity Response Was Higher Than Eukaryotes and Fungi in the Danjiangkou Reservoir.” Journal of Environmental Management 379: 124851. 10.1016/j.jenvman.2025.124851. 40056577

[emi470362-bib-0054] Webber, M. , B. Crow‐Miller , and S. Rogers . 2017. “The South–North Water Transfer Project: Remaking the Geography of China.” Regional Studies 51: 370–382. 10.1080/00343404.2016.1265647.

[emi470362-bib-0055] Worsfold, P. J. , P. Monbet , A. D. Tappin , M. F. Fitzsimons , D. A. Stiles , and I. D. McKelvie . 2008. “Characterisation and Quantification of Organic Phosphorus and Organic Nitrogen Components in Aquatic Systems: A Review.” Analytica Chimica Acta 624, no. 1: 37–58. 10.1016/j.aca.2008.06.016. 18706309

[emi470362-bib-0056] Wu, J. , H. Yang , R. D. Pancost , et al. 2021. “Variations in Dissolved O_2_ in a Chinese Lake Drive Changes in Microbial Communities and Impact Sedimentary GDGT Distributions.” Chemical Geology 579: 120348. 10.1016/j.chemgeo.2021.120348.

[emi470362-bib-0057] Wu, Y. , Y. Zhang , X. Yang , et al. 2022. “Deterministic Processes Shape Bacterial Community Assembly in a Karst River Across Dry and Wet Seasons.” Frontiers in Microbiology 13: 938490. 10.3389/fmicb.2022.938490. 36274723 PMC9584624

[emi470362-bib-0058] Xu, Z. , J. Huang , Z. Chu , et al. 2023. “Plant and Microbial Communities Responded to Copper and/or Tetracyclines in Mycorrhizal Enhanced Vertical Flow Constructed Wetlands Microcosms With *Canna indica* L.” Journal of Hazardous Materials 451: 131114. 10.1016/j.jhazmat.2023.131114. 36870129

[emi470362-bib-0059] Yan, Q. , J. C. Stegen , Y. Yu , et al. 2017. “Nearly a Decade‐Long Repeatable Seasonal Diversity Patterns of Bacterioplankton Communities in the Eutrophic Lake Donghu (Wuhan, China).” Molecular Ecology 26, no. 14: 3839–3850. 10.1111/mec.14151. 28437572

[emi470362-bib-0060] Yang, Q. , D. Li , W. Chen , et al. 2023. “Dynamics of Bacterioplankton Communities During Wet and Dry Seasons in the Danjiangkou Reservoir in Hubei, China.” Life (Basel) 13, no. 5: 1206. 10.3390/life13051206. 37240851 PMC10220977

[emi470362-bib-0061] Zhang, L. , C. Zhang , K. Lian , and C. Liu . 2021. “Effects of Chronic Exposure of Antibiotics on Microbial Community Structure and Functions in Hyporheic Zone Sediments.” Journal of Hazardous Materials 416: 126141. 10.1016/j.jhazmat.2021.126141. 34492930

[emi470362-bib-0062] Zhang, P. , J. Fu , and L. Hu . 2012. “Effects of Alkali Stress on Growth, Free Amino Acids and Carbohydrates Metabolism in Kentucky Bluegrass ( *Poa pratensis* ).” Ecotoxicology 21, no. 7: 1911–1918. 10.1007/s10646-012-0924-1. 22592662

[emi470362-bib-0063] Zhou, C. , Z. Wang , M. Ran , Y. Liu , and Z. Song . 2024. “Nano‐Selenium Ameliorates Microplastics‐Induced Injury: Histology, Antioxidant Capacity, Immunity and Intestinal Microbiota of Grass Carp ( *Ctenopharyngodon idella* ).” Ecotoxicology and Environmental Safety 285: 117128. 10.1016/j.ecoenv.2024.117128. 39342759

[emi470362-bib-0064] Zhu, L. , L. Feng , D. Zhang , et al. 2025. “Eukaryotic Plankton Community and Assembly Processes in a Large‐Scale Water Diversion Project in China.” Scientific Reports 15, no. 1: 4365. 10.1038/s41598-025-87983-9. 39910192 PMC11799226

[emi470362-bib-0065] Zuo, Y. , L. Li , T. Zhang , et al. 2010. “Contribution of Streptomyces in Sediment to Earthy Odor in the Overlying Water in Xionghe Reservoir, China.” Water Research 44, no. 20: 6085–6094. 10.1016/j.watres.2010.08.001. 20800260

