# Supplementary figures and images for: The Seasonal and Spatial Patterns of Planktonic Bacterial and Fungal Community Structure and the Response to Environmental Factors in the Danjiangkou Reservoir, China
Source: Environ Microbiol Rep. 2026 May 13;18(3):e70362. doi: 10.1111/1758-2229.70362 (PMC13169159; doi:10.1111/1758-2229.70362)

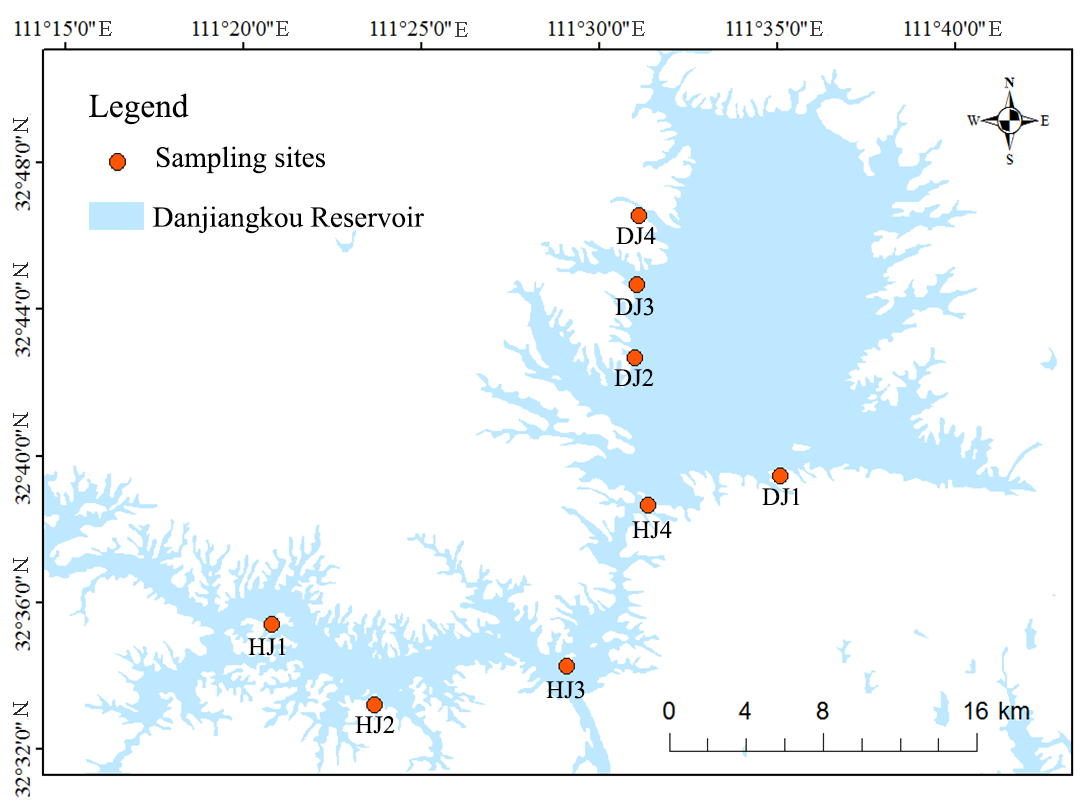

Supplement: Supplementary file 1 — Figure S1: Distribution of sampling sites in the DJKR: a total of 8 sampling sites (4 sampling sites at HJ and DJ respectively). [file EMI4-18-e70362-s004.tif]
